# The Risk of Venous Thromboembolism in Neuroendocrine Neoplasms

**DOI:** 10.3390/cancers15225477

**Published:** 2023-11-20

**Authors:** Monika Wójcik-Giertuga, Anna Malczewska-Herman, Beata Kos-Kudła

**Affiliations:** Department of Endocrinology and Neuroendocrine Tumors, Department of Pathophysiology and Endocrinology, Faculty of Medical Sciences in Zabrze, Medical University of Silesia, Ceglana 35, 40-514 Katowice, Poland; anna.malczewska@sum.edu.pl (A.M.-H.); bkoskudla@sum.edu.pl (B.K.-K.)

**Keywords:** venous thromboembolism, cancer-associated thrombosis, thrombosis, neuroendocrine neoplasms, thromboembolic risk

## Abstract

**Simple Summary:**

Malignant tumors are known to exhibit an increased risk of venous thromboembolism (VTE). Neuroendocrine neoplasms (NENs) represent a heterogeneous group that significantly differs from other malignancies in terms of biology and clinical management. The data on the incidence of thromboembolic events in NENs is limited and no specific recommendations on the use of antithrombotic prophylaxis in this group have been established. Such issues led to this work. The article provides a comprehensive overview of the current knowledge on the thromboembolic events in NENs which represents a prerequisite for improved assessment of the VTE risk in NENs and optimized selection of patients who will benefit most from antithrombotic prophylaxis.

**Abstract:**

Neuroendocrine neoplasms (NENs) differ from other malignancies in their ability to produce hormones and biogenic amines, as well as offer a better prognosis in well-differentiated tumors. There are no definite data on the occurrence of thromboembolic events in NENs and no recommendations regarding the use of antithrombotic prophylaxis in this group. Accurate assessment of the thromboembolic risk in NENs represents an important issue, in order to reduce morbidity and mortality due to complications of VTE. The aim of this work was to review the occurrence of thromboembolic events in NENs and the use of antithrombotic prophylaxis in this group. A total of 28 studies identified on PubMed were analyzed. NENs, especially of pancreatic primary, exhibit an increased thrombotic risk. Atypical VTE locations are quite common in NENs. Hormonally active NENs are associated with a significantly increased thromboembolic risk. Further studies in NENs are needed to evaluate the parameters of coagulation and fibrinolysis as predictive biomarkers for VTE complications.

## 1. Introduction

Venous thromboembolism (VTE) may present with deep vein thrombosis and/or pulmonary embolism. VTE is the second most common cause of death in cancer patients [1]. For years, cancer had been hypothesized as a significant factor in the development of thrombosis. Even 10–20% of patients with VTE have a history of malignancy [2]. One of the acute complications of VTE is primarily pulmonary embolism. Acute pulmonary embolism is fatal in 2–8% of patients [3]. In the presence of an atrial or ventricular septal defect, peripheral embolism or stroke may also be a complication of lower limb venous thrombosis [3]. Chronic complications of VTE occurring in almost 50% of patients include chronic venous insufficiency and post-thrombotic syndrome. Recurrent episodes of pulmonary embolism lead to the development of chronic thromboembolic pulmonary hypertension with poor prognosis [3]. Among the complications of VTE, thrombotic microangiopathy and disseminated intravascular coagulation (DIC) are less common [2]. When defining the causes of thrombosis, one should quote Virchow’s triad [4]. It describes the three most important risk factors for the development of thrombosis, such as damage to the vessel wall, blood flow disorders, and blood hyperviscosity resulting from abnormal blood composition [4]. Under normal conditions, both coagulation and fibrinolysis are in equilibrium. Damage to the vessel wall and dysfunction of the vascular endothelium leads to the loss of its anticoagulant properties and the reduction of the efficiency of the fibrinolysis process [5]. In the case of tumor cells, their ability to interact with host cells such as endothelial cells results in the loss of their anticoagulant function [2]. The process of the activation of these cells associated with hemostasis, either by adhesion or by means of cytokines, therefore increases the thrombotic risk [2]. In the case of plasma hemostasis, the activation of coagulation occurs mainly through the activation of the extrinsic pathway, which is dependent on tissue factor (TF)—this is the main initiator of blood coagulation in vivo [6,7]. Platelet plugs and fibrin are presented on the cancer’s surface, which can support local activation of the blood coagulation [2]. The cancer cells may express TF on their surface and may release TF-positive microparticles (MP-microparticles) into the bloodstream, which are connected with the site of vascular injury and may increase the risk of venous thrombosis [6,7,8].

**Figure 1:**
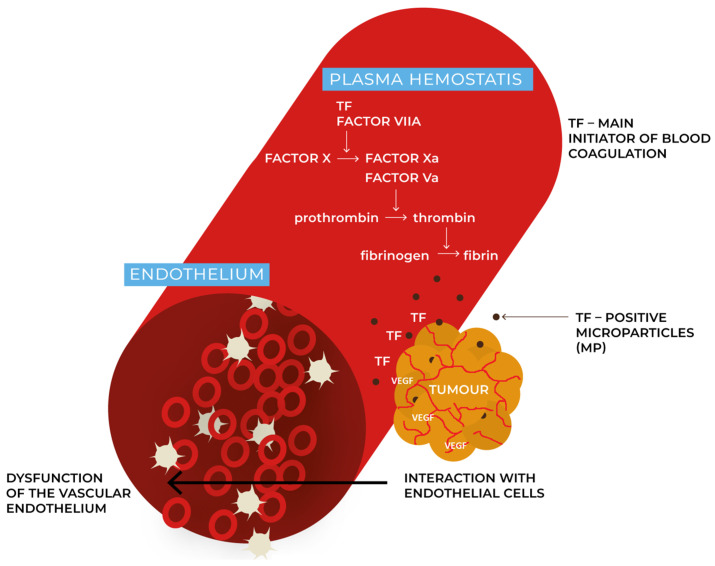
The potential mechanisms of the pathogenesis of thrombosis in NENs. The figure illustrates the role of endothelial and plasma hemostasis in thrombus formation in NENs. NENs can exhibit rich internal vascularity. Pro-angiogenic factors that occur in NENs can support local activation of blood coagulation. Dysfunction of the vascular endothelium can lead to the loss of its anticoagulant features and may cause the reduction of the efficiency of the fibrinolysis process. The activation of coagulation occurs mainly through the activation of the extrinsic pathway, which is especially dependent on tissue factor (TF)—the main initiator of blood coagulation in vivo. Cancer cells also may release TF-positive microparticles (MP-microparticles) into the bloodstream, which may increase the risk of venous thrombosis, but this requires further research in NENs. TF—tissue factor, VEGF—vascular endothelial growth factor.

Neuroendocrine neoplasms (NENs) represent a heterogeneous group of tumors, both in terms of biology and the ability to produce hormones and biogenic amines. NENs originate from the diffuse endocrine system (DES). GEP NENs (gastroenteropancreatic neuroendocrine neoplasms) which are located in the digestive system, constitute the majority (~70%) of all NENs and account for 2% of all malignant tumors of the gastrointestinal tract [9,10]. The small intestine and pancreas are the most common primary locations in the digestive system [9,10]. Based on the grading—the degree of differentiation and the degree of histological maturity (feature G), this group was divided into well-differentiated neuroendocrine neoplasms (NEN G1, NEN G2, and NEN G3) and neuroendocrine carcinomas (NEC) [9,10]. BPNENs (bronchopulmonary NENs) account for about 20–25% of all NENs [11]. NENs, especially of a pancreatic primary (10–30% of PNENs—pancreatic neuroendocrine neoplasms), can often be the source of ectopic secretion of hormones such as ACTH (adrenocorticotropic hormone), PTHrP (parathyroid hormone-related protein), GHRH (growth hormone releasing hormone), or vasopressin-causing syndromes of characteristic symptoms [9,10]. The most common syndrome associated with the overproduction of hormones in NENs is carcinoid syndrome, which is caused by excessive secretion of serotonin. It is most common in NENs of the small intestine [9,10]. NENs are, in general, characterized by slow growth and good prognosis. However, the location of the primary focus in the pancreas and large intestine is a prognostically unfavorable factor [9]. Neuroendocrine carcinomas (NECs) account for 10–20% of NENs and are also characterized by a worse prognosis compared to other NENs [9]. The pathogenesis of thrombosis in NENs remains unclear, although some potential mechanisms have been proposed. NENs exhibit a high expression of pro-angiogenic factors [12,13,14]. This process may play a role in tumor thrombosis formation, but it requires further research. Similarly, in functional NENs increased serotonin concentrations may lead to endothelial fibrosis and the dysfunction of the endothelium [15]. The potential mechanisms of the pathogenesis of thrombosis in NENs are demonstrated in Figure 1. The aim of this work was to review the occurrence of thromboembolic events in NENs and the use of antithrombotic prophylaxis in this group.

## 2. Methods

To identify all relevant studies on thrombosis in NENs, we undertook a literature search on PubMed (between 1992 and 2023). The search criteria included: “cancer-associated thrombosis”, “thrombosis AND neuroendocrine tumors”, “thrombosis AND neuroendocrine neoplasms”, and “venous thromboembolism AND neuroendocrine tumors”. Studies in other languages than English or Polish or those not related to the study goals were excluded. Overall, 56 studies were identified and included in the analysis. Amongst these, 28 articles (including case reports) presented venous thromboembolism in NENs (Table 1).

## 3. The Occurrence of VTE in Neuroendocrine Neoplasms

A history of malignancy constitutes at least a moderate risk factor for VTE [3]. It is increased by up to 4–7 times in cancer patients compared to the healthy population [43]. The risk factors for VTE also include the treatment (chemotherapy, radiotherapy, hormone therapy, surgical treatment, or the use of steroids or central catheters) [3]. According to Walker et al., in other cancers, the location of the primary tumor represents an important VTE risk factor, as well as the early implementation of the prophylaxis in patients at increased thromboembolic risk, preferably immediately after diagnosis [44]. An Italian retrospective study reported an increased risk of thrombosis in GEP NENs [19]. Among 160 patients over a median follow-up of 62 months (between 2000–2016), 12 NENs exhibited VTE events which comprised symptomatic deep vein thrombosis in 9, and pulmonary embolism in 3 (symptomatic in 2, and asymptomatic in 1) [19]. NENs with the pancreatic primary and those at the higher tumor grade were at the highest thromboembolic risk [19]. The majority of NENs (9/12, 75%) that developed VTE were of the pancreatic primary, the rest comprised two intestinal NENs and in one, the location of the primary tumor was unknown [19]. Five patients were in stage IV, 2 patients in stage III, 3 patients in stage II, and 2 patients in stage I [19]. In 10 patients, VTE developed after or concurrently at the time of NEN diagnosis (median 12 months), whereas in 2 patients VTE developed 4 and 6 months before diagnosis, respectively [19]. The thrombotic events most often occurred within two years from diagnosis, which is consistent with the general data in other cancers [19]. The risk in cancer patients is in particular increased in the first 3–6 months after the diagnosis [3]. However, it has been also suggested that the risk remains increased even up to 15 years after the diagnosis [43]. In 25% of patients (3/12), the thromboembolic event occurred in the perioperative period related to GEP NEN surgery, no cases of VTE for surgery unrelated to GEP NEN were reported [19]. The 5 patients who were not surgically treated received somatostatin analogs alone or in combination with chemotherapy, radioligand therapy (RLT), or targeted therapies (including everolimus and sunitinib). In 2 cases, VTE developed during SSA (somatostatin analogs) therapy, and in 1 case during SSA and everolimus therapy [19]. The prediction of the risk of VTE was evaluated by the Khorana scale (KS). Interestingly, none of the NENs affected by deep vein thrombosis or pulmonary embolism received the score corresponding with the “high” thrombotic risk (>2 in the KS score) [19]. Additionally, a few case reports confirm the relationship between the pancreatic primary and an increased risk of VTE [16,17,18,36,37,38,40,41,42]. In the case of a 58-year-old male with an advanced pancreatic neuroendocrine carcinoma with metastases to the peritoneum, an increased risk of VTE was documented. In this patient, blood coagulation parameters were significantly elevated, including an increase in the activation of the fragment of prothrombin 1+2 (F1+2) and significantly elevated values of fibrinogen and D-dimers. The authors linked the increased thrombotic risk to the increased thrombin generation and fibrinolysis alterations and suggested prophylactic use of low molecular weight heparin to decrease the likelihood of thrombotic events due to their selected inhibition of prothrombinase and reduce morbidity and mortality related to thromboembolic complications [41]. According to the literature, the incidence of VTE incidents is highest at the time of diagnosis of metastatic cancer [45]. D-dimer concentration correlates with the stage of cancer [46].

MEN-1 (Multiple Endocrine Neoplasia, Type 1) syndrome is associated with an increased risk of VTE [20], especially in cases with the NENs of the pancreatic primary [20]. The occurrence of VTE in MEN-1 in 42% was associated with the perioperative time related to abdominal surgery (3 subtotal/distal pancreatectomies, 3 Whipple procedures, 2 duodenectomies, 1 hysterectomy) and other types of surgery (such as a thoracotomy, a trans-sphenoidal surgery, an esophageal surgery, a total parathyroidectomy, and orthopedic surgeries) [20].

## 4. Atypical Locations of VTE in Neuroendocrine Neoplasms

Atypical thrombosis includes deep vein thrombosis of the upper limbs (brachial and subclavian veins), cerebral venous thrombosis, as well as abdominal venous thrombosis (portal, splenic, mesenteric, and renal veins, and hepatic venous thrombosis—Budd–Chiari syndrome) [3]. Atypical thrombosis was documented in NENs in a few locations. 

Development of portal, splenic, and mesenteric thrombosis [26] represents a significant clinical problem that increases mortality in cancer patients which was documented in hepatocellular carcinoma (HCC) or renal cell carcinoma [26,47,48]. Pancreatic tumors also often invade adjacent blood vessels [26]. Amongst NENs, an increased risk of portal vein thrombosis was reported in particular in PNENs [18]. Surgical series confirm that tumor thrombosis is linked to about 5% of PNENs [26], but this is often frequently underreported on pre-operative imaging examinations. Balachandran et al. suggested that hormonally inactive NENs are characterized by an enhanced addition of secreted venous thrombosis in the tumor area, which was confirmed by the assessment of CT scans in 33% of patients [32]. It is unclear which site exactly is favored for tumor thrombosis in PNENs. Most often the portal vein, followed by the splenic vein or superior mesenteric vein, was reported [26]. This is clinically relevant since the presence of tumor thrombosis leads to the development of portal hypertension, gastric varices, and the increased risk of very serious complications such as bleeding from the gastrointestinal tract, which significantly worsens a patient’s prognosis [26]. According to Moyana et al., PNENs are associated with an increased risk of splenic vein thrombosis and sinus portal hypertension (SPH—sinus portal hypertension) [28]. The study was a 12-year retrospective review of imaging findings to confirm the existence of splenic vein thrombosis and to investigate the association with SPH [28]. Out of 61 patients with PNENs, 8 were diagnosed with splenic vein thrombosis and gastric varices at the time of diagnosis [28]. All patients were non-functional NENs, with NEN G2 diagnosed in 7 cases, and NEN G3 in 1 case. The majority of patients (5/8, 62,5%) were at advanced disease stage (IV), two (2/8, 25%) were at stage III, and one (1/8, 12,5%) was at stage II [28]. All four patients with recognized SPH underwent surgical resection and the mean follow-up was 8.5 years when the symptoms did not occur [28].

In the case of a 62-year-old female diagnosed with a poorly differentiated pancreatic neuroendocrine carcinoma, dilatation of the portal, splenic, and superior mesenteric veins typical for thrombosis was confirmed [21]. In the 99mTc-octreotide whole-body scintigraphy study, uptake of the radiotracer in the epigastric region, typical of thrombosis localization, was described. The patient did not consent to surgical thrombectomy. Radioisotope treatment was administered (2 cycles of 177Lu-DOTA-TATE) and resulted in an improvement of the ascites; the thrombosis was stable [21]. The mechanism of thrombosis formation due to direct contact between the tumor and the vessel was concluded as worse prognostically than thrombosis associated with a generalized hypercoagulable state, as it responds worse to treatment [21]. Interestingly, features of thrombosis can be identified on functional imaging performed in NENs, such as 68Ga-DOTA-TATE PET/CT, which assesses the expression of somatostatin receptors in NENs. Several case reports documented the identification of portal or inferior mesenteric vein thrombosis on 68Ga-DOTA-TATE PET/CT in PNENs [23,34,35,39,49]. The pathogenesis of tumor thrombosis in NENs remains unclear. PNENs are often predisposed to the development of portal vein and vena cava thrombosis due to the direct infiltration of the tumor into the lumen of these vessels [21]. The etiology of this condition can be primary and secondary, associated with numerous cases, e.g., tumor pressure on the portal vein, inflammation, or anticancer treatment [18]. It is possible that pro-angiogenic factors play a role in promoting the development of tumor thrombosis, but it requires further investigation. It seems that one of the hypotheses is the relationship between NENs and the strong expression of pro-angiogenic factors and vascular endothelial growth factors (VEGF) [12,13,14,26]. This hypothesis has been proven in other cancers. An increased expression of both VEGF and platelet-derived endothelial cell growth factor (PD-ECGF) were identified in HCC with tumor thrombosis [26,50]. Moyana et al. associated the occurrence of splenic thrombosis with the slow growth of NENs and the rich internal vascularity characteristic of PNENs [28]. The presence of tumor thrombosis, especially in PNENs, should not disqualify patients from surgical management [28]. The authors emphasized that there is a benefit from the distal pancreatic resection for PNENs localized in the tail of the pancreas [28]. According to the ENETS (European Neuroendocrine Tumor Society) guidelines, in locally advanced PNENs associated with the tumor thrombus, radical surgical treatment is recommended except for the cases with the invasion of the portal vein system with portal cavernoma and invasion of the superior mesenteric artery [51]. Tian et al. demonstrated for the first time the efficacy of TACE (TACE—transcatheter arterial chemoembolization) and anticoagulation in the case of PNEN in a 50-year-old male with unresectable liver metastases and portal vein thrombosis [18].

Various methods of treating tumor thrombosis in PNENs have been described in the literature as summarized by Robertis et al. [26,29,30,36,37,38,40,42], including effective selective portal thrombectomy [26,36,37] or en-bloc resection of the clotted vein together with the section of the pancreas affected by the tumor [26,29,42] or selective resection of the venous section affected by the tumor [26,38,40]. According to Prakash et al., thrombectomy from the portal vein system is effective during pancreatectomy in selected advanced PNENs [30]. In a cohort of 245 PNENs undergoing surgical treatment, in 9 patients (3.8%) thrombectomy of the portal vein was performed. All nine patients were non-functional and eight patients (8/9, 89%) had tumors located in the body and tail of the pancreas [30]. The authors described a venotomy on the anterior surface of the vessel and the direct extraction of the thrombus followed by a closure venography [30].

NENs may develop Budd–Chiari syndrome which is caused by hepatic vein thrombosis that partially or completely blocks blood flow from the liver [15]. In the case of liver metastases, it is hypothesized that metastatic tumors may cause venous blood flow disorders and predispose one to the development of the syndrome [15]. Its incidence is approximately 1 in 2.5 million people [15]. It was documented in a 57-year-old female with a disseminated rectal NEN and obstruction of the venous outflow from the liver [15], as well as in two other cases of a mediastinal carcinoid and metastatic bronchial carcinoid [15]. The most common symptoms are ascites, abdominal pain, and hepatomegaly [15].

The internal jugular vein represents another rare localization of thrombosis. Thrombosis of the right subclavian vein was reported in a 52-year-old male who presented with cervical edema in the course of a lung large cell neuroendocrine carcinoma. The authors concluded that in case of atypical localization of the thrombosis, a malignant tumor of the lung or mediastinum should always be excluded [22]. The suggested potential mechanisms responsible for the development of thrombosis in this localization comprised the rapid blood flow in the SVC vessel, the production of important procoagulant/fibrinolytic factors and cytokines, the interaction of tumor cells with the hemostasis system, and tumor pressure on the SVC and blood stasis [22].

## 5. VTE in Hormonally Active Neuroendocrine Neoplasms

NENs may produce hormones or other biologically active substances and cause hormonal syndromes. The relationship between functional tumors and thrombosis remains unclear.

### 5.1. Carcinoid Syndrome

Hormonally active serotonin-producing NENs most often in the small intestine with metastases to the liver are associated with the development of carcinoid syndrome [9,52]. Elevated serotonin concentration leads to endothelial fibrosis, which may represent one of the thrombosis causative factors, however, this requires further study [15]. Hollander et al. reported that NENs with carcinoid syndrome and carcinoid heart disease are at increased risk of thrombosis [24]. Tricuspid regurgitation is one of the most common complications of carcinoid syndrome and often requires surgical treatment to replace the valve. A 59-year-old male diagnosed with a small intestinal NEN, two weeks after tricuspid valve replacement, developed thrombus at the site of the prosthetic heart valve. Prior to the procedure, serotonin levels exceeded the upper limit of the norm by almost 5 times. Increased levels of serotonin may represent a signal for the activation of platelets and, in this mechanism, lead to thrombosis. However, the high morbidity associated with tricuspid regurgitation also requires the implementation of appropriate management, hence the importance of appropriate perioperative management [24]. As it is known, NENs can cause various cardiac complications and, in addition to the above-mentioned tricuspid valve dysfunction, may cause thrombus formation in the right atrium of the heart as described in the case of a 74-year-old patient with a duodenal NEN with metastases to the liver and lymph nodes [27].

### 5.2. Glucagonoma

Glucagonoma represents a very rare pancreatic NEN (0.01–0.1/1 mln persons/year) [53]. It can be related to MEN-1 syndrome in 1–20% of cases [9]. Thromboembolic complications are identified even in 30–80% of pancreatic glucagonomas [17]. Its clinical manifestations include necrotic migratory erythema, diabetes, depression, weight loss, and deep venous thrombosis in 25% of patients [17,54]. It is associated with the typical presentation of deep vein thrombosis and pulmonary embolism [17]. In a 68-year-old patient, venous sinus thrombosis (CS VT) developed [17], which represents a rather unusual location of thrombosis in glucagonoma and may manifest primarily with a headache, and in 50% of cases with focal neurological deficits [17]. Early diagnosis of this tumor is crucial due to the disease’s aggressiveness—glucagonomas are malignant in ~80% of cases [17]. Massironi et al. reported two patients who developed VTE (deep vein thrombosis and pulmonary embolism) in functioning GEP NENs (glucagonoma and gastrinoma) [19].

### 5.3. Endogenous Cushing’s Syndrome

Lung NENs may secrete ACTH and cause ectopic hormonal syndromes, such as Cushing’s [25]. A meta-analysis by Wagner et al. identified that the ectopic Cushing’s syndrome is associated with a significantly increased risk of VTE due to increased von Willebrand factor (vWF), decreased clotting time (APTT), and increased coagulation factor VIII [55]. It is vital to monitor these parameters to consider the benefits of thromboprophylaxis in Cushing’s syndrome [55]. The ectopic Cushing’s syndrome was reported in a lung carcinoid with hepatic metastases [25]. Hypercoagulable syndrome complicated the primary disease diagnosis. Studies on the coagulation mechanisms identified increased activity of the von Willebrand factor, increased concentration of factor VIII, and decreased fibrinogen [25]. Ectopic ACTH secretion was found in a 78-year-old patient with pancreatic neuroendocrine carcinoma (Ki-67 approximately 40%) [16]. ACTH-secreting PNENs are a rare condition characterized by an aggressive course and significant mortality associated with increased thrombotic risk and hypercortisolism. The patient developed deep vein thrombosis of the lower limbs, as well as pulmonary embolism and disseminated intravascular coagulation (DIC) [16]. DIC is the most severe form of systemic activation of coagulation, which, in the case of cancer, is slightly less severe than in sepsis or trauma, but leads to the consumption of blood coagulation factors and platelets and can manifest as bleeding at the site of the tumor. DIC is more common in acute lymphoblastic leukemia (15–20% of patients) compared to solid tumors (7% of patients) [2]. Nakano et al. demonstrated, based on advanced lung cancer, that coagulation disorders due to DIC develop as a result of chronic inflammation and are mainly associated with changes in the concentrations of factors such as thrombomodulin and antithrombin [56]. DIC has been documented in pancreatic, recurrent NENs. It was first identified after the tumor biopsy and then after the initiation of carboplatin and etoposide chemotherapy. At the time of the second recurrence, DIC resulted in severe bleeding and, despite intensive treatment, was fatal [33].

**Figure 2:**
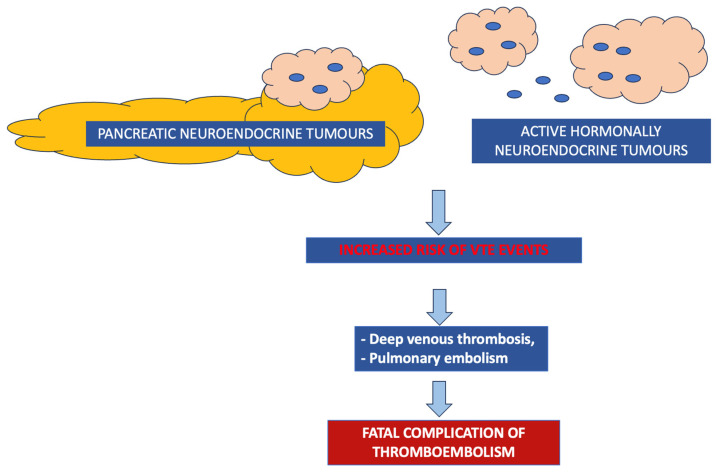
Groups of neuroendocrine neoplasms that exhibit an increased thromboembolic risk and the types of thromboembolic events in NENs. VTE—venous thromboembolism.

The graphical presentation of selected groups of NENs at increased thromboembolic risk and types of thromboembolic events is presented in Figure 2. The summary of the incidence of thromboembolism events in NENs is presented in Table 1.

## 6. Antithrombotic Prophylaxis in Neuroendocrine Neoplasms

There are no separate recommendations on the use of antithrombotic prophylaxis in NENs. Further studies are needed to establish its role in NENs. 

## 7. Conclusions and Future Directions

NENs of either the digestive or respiratory systems are characterized by an increased risk of thrombosis. Atypical locations of VTE are quite common in NENs. Hormonally active NENs are associated with a significantly increased VTE risk. The highest risk of thrombosis in NENs is associated with the pancreatic primary and the high tumor grade. Selection of NENs with an increased risk of VTE for implementation of thromboprophylaxis is crucial to reduce morbidity and mortality due to VTE complications. Further studies to unravel the potential mechanisms of thrombosis in NENs, refine the thromboembolic risk assessment, and evaluate coagulation and fibrinolysis parameters as prognostic and predictive biomarkers for VTE complications are vital to improving NEN patients’ prognosis and survival.

## Figures and Tables

**Table 1:** The summary of the incidence of thromboembolism events in NENs.

No.	Author, Year	Primary Tumor Localization	Pathology	TNM Stage	Functionality Status	Number of Patients with VTE (% VTE)	Type of Thromboembolic Complications	References
**1**	**Yoshihara et al. (2022)**	Pancreatic	NEC (Ki-67 40%)	N/A	F: Ectopic Cushing’s syndrome (ectopic ACTH syndrome)	1/1 (CR)	**VTE** (deep vein thrombosis, pulmonary embolism) **DIC**	[16]
**2**	**Delli Colli et al. (2022)**	Pancreatic	G1 (Ki-67 2%)	I	F: Glucagonoma	1/1 (CR)	**VTE** (cerebral sinus venous thrombosis)	[17]
**3**	**Tian et al. (2022)**	Pancreatic	N/A	IV	N/A	1/1 (CR)	**VTE** (portal vein thrombosis)	[18]
**4**	**Massironi et al. (2021)**	Pancreatic: 9/12 (75%) Small intestinal: 2/12 (16.7%) Unknown: 1/12 (8.3%)	G1: 3/12 (25%) G2: 6/12 (50%) G3: 2/12 (16.7%) N/A: 1/12	I: 2/12 (16.7%) II: 3/12 (25%) III: 2/12 (16.7%) IV: 5/12 (41.7%)	NF: 10/12 (83.33%), F: 2/12 (16.67%)	12/160 (7.5%)	**VTE** (deep venous thrombosis: 9/12 (75%), pulmonary embolism: 3/12 (25%))	[19]
**5**	**Lee et al. (2021)**	Pancreatic: 29/36 (80%)	N/A	7/36 metastatic (19.4%)	NF: 22/36 (75.9%) F: Insulinoma 7/36 (24%) Gastrinoma and/or Zollinger–Ellison 16/36 (44.4%) Glucagonoma (None)	36/286 MEN-1 pts (12.9%)	**VTE** (deep venous thrombosis, pulmonary embolism)	[20]
**6**	**Amoui et al. (2021)**	Pancreatic	NEC	IV	N/A	1/1 (CR)	**VTE** (portal vein tumor thrombus)	[21]
**7**	**Liaqat et al. (2021)**	Pulmonary	NEC	N/A	N/A	1/1 (CR)	**VTE** (right internal jugular vein (IJV) thrombosis and right subclavian vein thrombosis)	[22]
**8**	**Zhu et al. (2020)**	Pancreatic	N/A	N/A	N/A	1 (CR)	**VTE** (portal vein tumor thrombus)	[23]
**9**	**Hollander et al. (2019)**	Small intestinal	N/A	Metastatic disease	F: Carcinoid syndrome	1/1 (CR)	Bioprosthetic valve thrombosis	[24]
**10**	**Yang et al. (2019)**	Bronchial	Typical carcinoid	IV	F: Ectopic Cushing’s syndrome (ectopic ACTH syndrome)	1/1 (CR)	**VTE** (pulmonary embolism)	[25]
**11**	**De Robertis et al. (2018)**	Pancreatic	G2: 4/6 (66.7%) G3: 2/6 (33.3%)	N/A	N/A	6/6 (CR)	**VTE** (tumor thrombus)	[26]
**12**	**Yu (2018)**	Duodenal	G2 (Ki-67 5–10%)	Metastatic	N/A	1/1 (CR)	**VTE** (right atrial tumor thrombus)	[27]
**13**	**Moyana et al. (2017)**	Pancreatic	G2: 7/8 (87.5%) G3: 1/8 (12.5%)	II: 1/8 (12.5%) III: 2/8 (25%) IV: 5/8 (62.5%)	NF: 8/8 (100%)	8/61 (13.11%)	**VTE** (splenic vein thrombosis and gastric varices in CT imaging)	[28]
**14**	**Rodriguez et al. (2014)**	Pancreatic	G2	II	NF	1/1 (CR)	**VTE** (portal vein tumor thrombus)	[29]
**15**	**Prakash et al. (2015)**	Pancreatic	G2: 4/9 (44.4%) G3: 1/9 (11.1%) N/A: 4/9	II: 7/9 (77.8%) IV: 2/9 (22.2%)	NF: 9/9 (100%)	26/245 (11%) 9 pts (3.8%) underwent portal venous tumor thrombectomy	**VTE** (portal vein tumor thrombus)	[30]
**16**	**Hurtado-Cordovi et al. (2013)**	Rectal	N/A	IV	N/A	1/1 (CR)	**VTE** (Budd–Chiari syndrome)	[15]
**17**	**Busch et al. (2013)**	Pulmonary	Small cell NEC	IV	F: SIADH	1/1 (CR)	**VTE** (pulmonary embolism)	[31]
**18**	**Balachandran et al. (2012)**	Pancreatic	N/A	N/A	NF: 29/29 (100%)	29/88 (33%)	**VTE** (tumor thrombus)	[32]
**19**	**Teh RW et al. (2012)**	Pancreatic (probably)	NEC	N/A	NF	1/1 (CR)	**DIC**	[33]
**20**	**Naswa et al. (2012)**	Pancreatic	G2 (Ki-67 4%)	N/A	NF	1/1 (CR)	**VTE** (tumor thrombus)	[34]
**21**	**Lim et al. (2011)**	Pancreatic	N/A	IV	N/A	1/1 (CR)	**VTE** (portal vein tumor thrombus)	[35]
**22**	**Tsuchikawa et al. (2011)**	Pancreatic	N/A	N/A	N/A	1/1 (CR)	**VTE** (portal vein tumor thrombus)	[36]
**23**	**Barbier et al. (2010)**	Pancreatic	G2 (Ki-67 3%)	IV	NF	1/1 (CR)	**VTE** (portal vein tumor thrombus)	[37]
**24**	**Kawakami et al. (2006)**	Pancreatic	NEC	N/A	NF	1/1 (CR)	**VTE** (portal vein tumor thrombus)	[38]
**25**	**Nguyen (2005)**	Pancreatic	N/A	N/A	N/A	1/1 (CR)	**VTE** (portal vein tumor thrombus)	[39]
**26**	**Bedirli et al. (2004)**	Pancreatic	Islet cell carcinoma	N/A	NF	1/1 (CR)	**VTE** (portal vein tumor thrombus)	[40]
**27**	**Di Micco et al. (2002)**	Pancreatic	N/A	IV	N/A	1/1 (CR)	**VTE**	[41]
**28**	**Watase et al. (1992)**	Pancreatic	Islet cell carcinoma	N/A	NF	1/1 (CR)	**VTE** (tumor thrombus)	[42]

CR—case report, DIC—disseminated intravascular coagulation, NEC—neuroendocrine carcinoma, F—functional, NF—Non-Functional, N/A—not available, SIADH—syndrome of inappropriate antidiuretic hormone secretion, VTE—venous thromboembolism.
